# Dyslipidemia and hyperuricemia: a cross-sectional study of residents in Wuhu, China

**DOI:** 10.1186/s12902-023-01528-7

**Published:** 2024-01-02

**Authors:** Yicheng Fang, Wendan Mei, Chenxu Wang, Xia Ren, Jian Hu, Fan Su, Lei Cao, Grace Tavengana, Mingfei Jiang, Huan Wu, Yufeng Wen

**Affiliations:** 1 School of Public Health, Wannan Medical College, 22 West Wenchang Road, 241002 Wuhu, Anhui Province China; 2 School of Clinical Medicine, Wannan Medical College, 22 West Wenchang Road, 241002 Wuhu, Anhui Province China; 3 School of Laboratory Medicine, Wannan Medical College, 22 West Wenchang Road, 241002 Wuhu, Anhui Province China

**Keywords:** Hyperuricemia, Dyslipidemia, Clinical classification, Components

## Abstract

**Background:**

While dyslipidemia has been recognized as a potential risk factor for hyperuricemia, there is currently a dearth of large-scale data specifically focused on studying the relationship between these two conditions. To address this gap, the present study analyzed a dataset of 298,891 physical examination records to investigate in greater detail the clinical classification and compositional relationship between hyperuricemia and dyslipidemia.

**Methods:**

For this investigation, a cross-sectional research design was utilized to analyze physical examination data that was gathered from Yijishan Hospital in Wuhu, China between 2011 and 2016. Logistic regression was employed to examine the association between hyperuricemia and dyslipidemia. Furthermore, the association between hyperuricemia and dyslipidemia was evaluated based on the clinical classifications of dyslipidemia and its components.

**Results:**

A total of 298,891 participants from China (124,886 [41.8%] females) were included in the study, with an age range of 18 to 90 years (mean [SD]: 47.76 [13.54] years). In multivariate analysis, the odds of hyperuricemia was 1.878 times higher in patients with dyslipidemia compared to those without dyslipidemia (95% confidence interval [CI]: 1.835–1.922). In the clinical classification of dyslipidemia, individuals with hypertriglyceridemia and mixed hyperlipidemia had 1.753 times (95% CI: 1.706–1.802) and 1.925 times (95% CI: 1.870–1.982) higher odds of hyperuricemia, respectively, compared to those without dyslipidemia. Among the components of dyslipidemia, the odds ratios for hyperuricemia in individuals in the fourth quartile compared to those in the first quartile were 3.744 (95% CI: 3.636–3.918) for triglycerides, 1.518 (95% CI: 1.471–1.565) for total cholesterol, and 1.775 (95% CI: 1.718 − 1.833) for non-high-density lipoprotein cholesterol.

**Conclusions:**

Dyslipidemia has been independently linked with hyperuricemia. Moreover, the elevation of triglycerides or total cholesterol levels, including conditions such as hypertriglyceridemia and mixed hyperlipidemia, have been observed to have a positive association with the development of hyperuricemia.

## Introduction

Hyperuricemia is a common metabolic disorder [1] caused by excessive uric acid production from factors such as a purine-rich diet, alcohol consumption, obesity, or reduced uric acid excretion due to conditions such as renal insufficiency, diabetes, and hypertension, among others [2, 3]. The resultant increase in blood uric acid levels exceeds the normal range of the human body. In recent decades, the incidence of hyperuricemia has increased rapidly worldwide [4]. This not only adversely affects individual health [5] but also places a significant burden on medical systems globally [6].

Numerous epidemiological studies have demonstrated that unhealthy lifestyle habits, such as smoking and drinking, can increase the risk of hyperuricemia [7, 8]. Additionally, hyperuricemia has been associated with various diseases, such as dyslipidemia, obesity, hypertension, and diabetes [9–12]. Among these, dyslipidemia is a common disorder of lipoprotein metabolism in humans [13], and its main components include total cholesterol(TC), triglyceride(TG), high-density lipoprotein cholesterol(HDL-C), and low-density lipoprotein cholesterol(LDL-C) [14].

There exists ample evidence indicating a significant association between dyslipidemia and an increased risk of hyperuricemia [15]. However, the association between components of dyslipidemia and hyperuricemia remains controversial. Cross-sectional studies have found a positive association between hyperuricemia and elevated levels of TG, TC, and LDL-C, and a negative association with decreased levels of HDL-C [16–18]. At the same time, we found that few studies have examined the relationship between dyslipidemia and hyperuricemia from the perspective of clinical classification of dyslipidemia. From a physiological and metabolic standpoint, dyslipidemia might escalate the risk of hyperuricemia by triggering or intensifying processes such as insulin resistance, inflammatory responses, and purine metabolic disturbances [19, 20]. There could be an interplay and mutual influence among these processes, forming a complex network that collectively propels the concurrent onset and progression of dyslipidemia and hyperuricemia. Therefore, we explored the relationship between hyperuricemia and dyslipidemia from two different perspectives of clinical types and components through a large amount of data.

## Materials and methods

### Study population

A cross-sectional study was conducted on the physical examination data of participants aged 18 to 90 from 2011 to 2016. Data provided by the First Affiliated Hospital of Wannan Medical College, Wuhu, China. In this study, subjects had to be at least 18 years of age and had no prior medical intervention for hyperuricemia. At the same time, complete data measurement and subject consent can be obtained. A total of 434,847 people participated. According to these criteria, the final physical examination data of 298,891 participants (174,005 men and 124,886 women) were included in this study.

### Anthropometric and laboratory measurements

We used questionnaires to record basic anthropometric data such as age, sex, smoking, and drinking habits. Subjects were wearing light clothing and no shoes. The height measurement accuracy is 0.1 cm, and the weight measurement accuracy is 0.1 kg. Body Mass Index (BMI) is accurate to 0.01 kg/m^2^. We measured systolic and diastolic blood pressure using a benchtop mercury sphygmomanometer.

Venous blood was drawn from subjects in the morning on an empty stomach, and all samples were analyzed within 24 hours. Serum uric acid (SUA), total cholesterol (TC), triglyceride (TG), high-density lipoprotein cholesterol (HDL-C), and low-density lipoprotein cholesterol (LDL-C) were detected by an automatic biochemical analyzer. In addition to these measurements, non-high-density lipoprotein (non-HDL) cholesterol was calculated as the difference between TC and HDL-C.

Fasting blood glucose (FBG), alanine aminotransferase (ALT), aspartate aminotransferase (AST), blood urea nitrogen (BUN), and serum creatinine (SCR) were also measured using the same instrument. All measurements were taken by a professional doctor and checked again to confirm the accuracy of the results.

### Diagnostic criteria

Hyperuricemia was defined as SUA > 420 mmol/L (male) or SUA > 360 mmol/L (female) [21, 22], which is a widely accepted diagnostic criterion. Subjects are considered to have dyslipidemia if they meet one of the following criteria at the time of examination: TC ≥ 5.2 mmol/L, TG ≥ 1.7 mmol/L, HDL-C < 1.0 mmol/L, LDL-C ≥ 3.4 mmol/L [23]. According to the clinical classification criteria [24, 25], dyslipidemia can be divided into the following four main categories: hypercholesterolemia, elevated TC level; hypertriglyceridemia, elevated TG level; mixed hyperlipidemia, TC, TG levels increased; low high-density lipoprotein cholesterol (HDL) dyslipidemia, decreased level of HDL-C.

Diabetes was defined as FBG ≥ 7.0 mmol/L [26]. Hypertension was defined as having a systolic blood pressure (SBP) ≥ 140 mmHg and/or diastolic blood pressure (DBP) ≥ 90 mmHg, or currently being on treatment for hypertension [27]. According to the Chinese body mass index classification standard (WS/T 428–2013), obesity is defined as BMI ≥ 28 kg/m^2^.

### Ethics statement

This study was approved by the Ethics Committee of Wannan Medical College. Since the subjects in this study came from people who underwent routine physical examination, only the oral and informed consent of the subjects was required with the approval of the ethics committee. Before the investigation, an oral informed consent form was provided to each subject.

### Statistical analysis

All data were analyzed using IBM SPSS Statistics Version 26. Categorical variables were presented as percentages and continuous variables as mean ± standard deviation. The chi-square test and t-test were employed to compare the differences between the hyperuricemia and normal groups. Binary logistic regression analysis was conducted to examine the association between the studied parameters and hyperuricemia. In the multivariate analysis, stepwise regression was utilized to filter and adjust for covariates. To investigate the relationship between dyslipidemia parameters and hyperuricemia in detail, hyperuricemia was used as the dependent variable, while the composition and clinical type of dyslipidemia were considered independent variables. Data were tested using an asymptotic model. Model 1 was unadjusted, Model 2 was adjusted for age and sex, and Model 3 was adjusted for age, sex, smoking, drinking, obesity, hypertension, diabetes, and ALT, AST, BUN, and SCR (fully adjusted model). Results were considered significant at a p-value < 0.05.

## Results

The baseline characteristics of study participants according to hyperuricemia are in Table 1. All subjects were divided into two groups. Compared with people with normal uric acid levels, the individuals in the hyperuricemia group were 5.1% higher for mean age, 9.1% higher for BMI, 6.3% higher for SBP, 5.2% higher for DBP, 5.0% higher for TC and 51.7% higher for TG. In line with these results, the rate of dyslipidemia was 52.2% higher, the rate of diabetes was 162% higher, the rate of hypertension was 77.6% higher and the rate of diabetes was 18.7% higher.

**Table 1:** Baseline characteristics of study participants according to hyperuricemia

Characteristic	Normal	Hyperuricemia	*t*/*x*^*2*^	*P*
Participants,N	254,105	44,786		
Age,mean ± SD,y	47.40 ± 13.27	49.81 ± 14.83	-32.23	< 0.0001
Sex,N(%)			11599.66	< 0.0001
Women	116,538(93.32)	8348(6.68)		
Men	137,567(79.06)	36,438(20.94)		
Smoking,N(%)	64,458(25.37)	16,495(36.83)	2533.83	< 0.0001
Drinking,N(%)	78,327(30.82)	21,668(48.38)	5271.59	< 0.0001
BMI,mean ± SD,kg/m^2^	23.48 ± 3.14	25.61 ± 3.17	-131.01	< 0.0001
Obesity,N(%)	20,224(7.96)	9343(20.86)	7111.07	< 0.0001
BP,mean ± SD,mmHg				
Systolic	118.21 ± 16.59	125.65 ± 17.15	-85.06	< 0.0001
Diastolic	76.82 ± 9.79	80.85 ± 10.24	-77.22	< 0.0001
Hypertension,N(%)	43,016(16.93)	13,461(30.06)	4281.79	< 0.0001
Diabetes,N(%)	11,816(4.65)	2472(5.52)	63.25	< 0.0001
Lipids,mean ± SD,mmol/L				
TC	4.60 ± 0.88	4.83 ± 0.97	-46.75	< 0.0001
HDL-C	1.39 ± 0.35	1.27 ± 0.31	73.26	< 0.0001
LDL-C	2.56 ± 0.76	2.59 ± 0.86	-8.33	< 0.0001
TG	1.45 ± 1.11	2.20 ± 1.65	-91.61	< 0.0001
Dislipidemia,N(%)	115,484(45.45)	30,986(69.19)	8586.50	< 0.0001
ALT,mean ± SD,(U/L)	25.61 ± 24.98	36.70 ± 30.63	-72.52	< 0.0001
AST,mean ± SD,(U/L)	22.77 ± 13.99	26.93 ± 16.17	-51.09	< 0.0001
BUN,mean ± SD,(mmol/L)	5.24 ± 1.39	5.66 ± 1.67	-50.40	< 0.0001
SCR,mean ± SD,(umol/L)	68.11 ± 17.66	81.25 ± 26.90	-99.66	< 0.0001

Abbreviations: BMI,body mass index ;BP,blood pressure; TC, total cholestero; TG, triglycerides; HDL-C, high-density lipoprotein cholesterol; LDL-C, low-density lipoprotein cholesterol; ALT, alanine transaminase; AST, aspartate transaminase; BUN, blood urea nitrogen; SCR, serum creatinine

Parameters associated with hyperuricemia in the binary logistic analysis of all study participants are in Table 2. In multivariate analysis, we found that dyslipidemia (OR, 1.878; 95% CI, 1.835–1.922), obesity (OR, 1.956; 95% CI, 1.898–2.015), and hypertension (OR, 1.368; 95% CI, 1.334–1.404) were independently and positively associated with hyperuricemia.

**Table 2:** Determinants of hyperuricemia using binary logistic analysis in all study participants

Parameters	Univariable		Multivariable	
	OR(95%CI)	*P*	OR(95%CI)	*P*
Age (per 1 year)	1.013(1.012–1.014)	< 0.0001	1.004(1.003–1.005)	< 0.0001
Male (vs. female)	3.697(3.605–3.790)	< 0.0001	1.317(1.270–1.366)	< 0.0001
Smoking	1.715(1.679–1.752)	< 0.0001	0.928(0.907–0.952)	< 0.0001
Drinking	2.103(2.061–2.147)	< 0.0001	1.132(1.105–1.160)	< 0.0001
Obesity	3.049(2.968–3.132)	< 0.0001	1.956(1.898–2.015)	< 0.0001
Hypertension	2.109(2.061–2.157)	< 0.0001	1.368(1.334–1.404)	< 0.0001
Diabetes	1.198(1.146–1.253)	< 0.0001	0.746(0.711–0.784)	< 0.0001
Dislipidemia	2.695(2.638–2.754)	< 0.0001	1.878(1.835–1.922)	< 0.0001
ALT (per 1 µ/L)	1.015(1.015–1.016)	< 0.0001	1.007(1.007–1.008)	< 0.0001
AST (per 1 µ/L)	1.022(1.021–1.022)	< 0.0001	1.009(1.008–1.009)	< 0.0001
BUN (per 1 mmol/L)	1.207(1.199–1.215)	< 0.0001	1.041(1.033–1.050)	< 0.0001
SCR (per 1 umol/L)	1.043(1.042–1.044)	< 0.0001	1.032(1.031–1.033)	< 0.0001

As detailed in Table 3, in the fully adjusted model, individuals in the fourth quartile of TG (OR, 3.774; 95% CI, 3.636–3.916), TC (OR, 1.518; 95% CI, 1.471–1.565), and non-HDL (OR, 1.775; 95% CI, 1.718 − 1.833) were more likely to have hyperuricemia than those in the first quartile. Conversely, higher quartiles of HDL-C (OR, 0.676; 95% CI, 0.653-0.700) and LDL-C (OR, 0.896; 95% CI, 0.870–0.923) were associated with a lower likelihood of hyperuricemia.

**Table 3:** Association of HUA with TG, TC, HDL-C, LDL-C, and Non HDL quartiles

Variable	OR (95% CI)		
	Model 1	Model 2	Model 3
TC			
Q1( < = 4.00)	1 [Reference]	1 [Reference]	1 [Reference]
Q2(4.01–4.56)	1.209(1.172–1.247)	1.163(1.127-1.200)	1.117(1.081–1.154)
Q3(4.57–5.19)	1.407(1.366–1.450)	1.300(1.261–1.341)	1.198(1.161–1.237)
Q4( > = 5.20)	1.905(1.851–1.960)	1.752(1.701–1.805)	1.518(1.471–1.565)
P value for trend	< 0.0001	< 0.0001	< 0.0001
TG			
Q1( < = 0.87)	1 [Reference]	1 [Reference]	1 [Reference]
Q2(0.88–1.25)	1.757(1.690–1.826)	1.501(1.443–1.561)	1.404(1.349–1.461)
Q3(1.26–1.86)	3.149(3.037–3.266)	2.446(2.357–2.538)	2.140(2.060–2.223)
Q4( > = 1.87)	6.416(6.199–6.640)	4.573(4.414–4.737)	3.774(3.636–3.916)
P value for trend	< 0.0001	< 0.0001	< 0.0001
HDL-C			
Q1( < = 1.12)	1 [Reference]	1 [Reference]	1 [Reference]
Q2(1.13–1.33)	0.810(0.789–0.831)	0.904(0.881–0.928)	0.999(0.972–1.027)
Q3(1.34–1.58)	0.581(0.565–0.597)	0.744(0.723–0.766)	0.887(0.860–0.914)
Q4( > = 1.59)	0.364(0.353–0.376)	0.534(0.517–0.552)	0.676(0.653-0.700)
P value for trend	< 0.0001	< 0.0001	< 0.0001
LDL-C			
Q1( < = 2.04)	1 [Reference]	1 [Reference]	1 [Reference]
Q2(2.05–2.52)	0.867(0.842–0.893)	0.844(0.820–0.870)	0.825(0.800-0.851)
Q3(2.53–3.03)	0.942(0.916–0.970)	0.865(0.840–0.891)	0.816(0.792–0.841)
Q4( > = 3.04)	1.140(1.109–1.172)	1.015(0.986–1.044)	0.896(0.870–0.923)
P value for trend	< 0.0001	< 0.0001	< 0.0001
Non HDL			
Q1( < = 1.12)	1 [Reference]	1 [Reference]	1 [Reference]
Q2(1.13–1.33)	1.443(1.396 − 1.491)	1.278(1.236 − 1.321)	1.192(1.152 − 1.234)
Q3(1.34–1.58)	1.894(1.835 − 1.955)	1.544(1.495 − 1.595)	1.342(1.298 − 1.387)
Q4( > = 1.59)	2.838(2.754 − 2.925)	2.214(2.146 − 2.283)	1.775(1.718 − 1.833)
P value for trend	< 0.0001	< 0.0001	< 0.0001

Model 1:Unadjusted

Model 2: Adjusted for age and sex

Model 3: Adjusted for age, sex, smoking, drinking, obesity, hypertension, diabetes, ALT, AST, BUN, SCR

The association between hyperuricemia and dyslipidemia in different clinical categories is in Table 4. In the fully adjusted model, we found significant associations between hyperuricemia and both hypertriglyceridemia (OR, 1.753; 95% CI, 1.706–1.802) and mixed hyperlipidemia (OR, 1.925; 95% CI, 1.870–1.982) in the clinical classification of dyslipidemia.

**Table 4:** Association between hyperuricemia and dyslipidemia in different clinical classification

Clinical type	*β*	*SE*	*WaldX* ^2^	*P*	OR(95%CI)
I					
Model 1	-0.348	0.017	419.613	< 0.0001	0.706(0.683–0.730)
Model 2	-0.289	0.018	273.077	< 0.0001	0.749(0.724–0.775)
Model 3	-0.258	0.018	204.343	< 0.0001	0.773(0.746–0.801)
II					
Model 1	0.779	0.013	3559.724	< 0.0001	2.178(2.123–2.235)
Model 2	0.625	0.013	2189.198	< 0.0001	1.868(1.820–1.918)
Model 3	0.562	0.014	1623.596	< 0.0001	1.753(1.706–1.802)
III					
Model 1	0.935	0.014	4671.861	< 0.0001	2.547(2.479–2.616)
Model 2	0.799	0.014	3234.758	< 0.0001	2.223(2.163–2.285)
Model 3	0.655	0.015	1950.771	< 0.0001	1.925(1.870–1.982)
IV					
Model 1	-0.141	0.023	36.557	< 0.0001	0.868(0.829–0.909)
Model 2	-0.405	0.025	270.189	< 0.0001	0.667(0.635-0.700)
Model 3	-0.410	0.025	276.740	< 0.0001	0.663(0.632–0.696)

Notes: I: Hypercholesterolemia II: Hypertriglyceridemia III: Mixed hyperlipidemia IV: low high-density lipoprotein cholesterol (HDL) dyslipidaemia

Model 1: Unadjusted

Model 2: Adjusted for age and sex

Model 3: Adjusted for age, sex, smoking, drinking, obesity, hypertension, diabetes, ALT, AST, BUN, SCR

## Discussion

To our knowledge, this is the most extensive cross-sectional study to date examining the relationship between hyperuricemia and various dyslipidemias. We have identified a significant correlation between dyslipidemia and hyperuricemia, particularly with hypertriglyceridemia and mixed hyperlipidemia. Elevated levels of TG and TC are significantly associated with an increased likelihood of hyperuricemia. Additionally, our analysis included non-high-density lipoprotein cholesterol, providing a more comprehensive evaluation of the dyslipidemic profiles associated with hyperuricemia.

Our results suggest that in addition to dyslipidemia, obesity, hypertension, and alcohol consumption are also significantly associated with hyperuricemia. These findings align well with observations from both cross-sectional analyses and prospective cohort studies [28–31]. Our finding that smoking and diabetes are associated with lower odds of hyperuricemia may contradict common sense. Upon examining the relevant data, we found some studies showed an inverse association between smoking and hyperuricemia, but only in women [32]. This may be because smoking causes oxidative stress, which leads to a drop in uric acid levels [33]. At the same time, the data showed that men with diabetes were less likely to develop hyperuricemia [34]. However, due to the limitations of the research method, we could not draw more specific conclusions. In addition, indicators of liver function (ALT, AST) and renal function (BUN, SCR) were positively associated with an increased likelihood of hyperuricemia [35, 36]. This may be due to food intake and the production of purine compounds, which the body breaks down in the liver, uric acid, which makes up the majority of it, is excreted through the kidneys. Elevation of these indicators indicates that the liver and kidney function of the human body is damaged, and the human body cannot decompose or excrete uric acid normally, resulting in blood uric acid in the human body exceeding the normal value [37].

A considerable body of cross-sectional epidemiological evidence indicates a significant positive association between the likelihood of hyperuricemia and levels of TG and TC, but a negative association with levels of HDL-C [15, 18, 38]. Our research also confirms this point, highlighting a noteworthy relationship where higher levels of TG are often accompanied by lower levels of HDL-C. This inverse association is in line with the established understanding that dyslipidemic profiles characterized by high TG typically present with reduced HDL-C, an indicator of altered lipid metabolism that is frequently observed in metabolic disorders. In recognizing the multifaceted nature of lipid profiles, we note the complexity in the association with total cholesterol, which is composed of HDL-C, LDL-C, and the cholesterol carried by TG in very low-density lipoprotein(VLDL). Although our current study did not delve into the intricacies of this relationship, it is an important aspect for future investigations to explore, particularly in relation to hyperuricemia. At the same time, we also found that TG is more related than other lipid components, which is consistent with the results of Hou et al. [9, 39]. Furthermore, a multicentric longitudinal study also observed that hypertriglyceridemia and low HDL-C concentrations are associated with elevated serum uric acid concentrations, further strengthening the links established in our study [20]. However, the specific mechanism of TG level and hyperuricemia has not been elucidated. Some studies have suggested that triglycerides can cause metabolic disorders of free fatty acids, accelerate the decomposition of adenosine triphosphate and lead to the increase of uric acid [9, 40]. Corroborating this, the prospective cohort study conducted by Chien and colleagues indicates an association between uric acid production and purine metabolism within the biosynthesis pathway of triglycerides [19]. Delving deeper into the mechanistic aspect, dyslipidemia can instigate the overactivity of xanthine oxidoreductase (XOR), a crucial enzyme in purine metabolism. This not only escalates the production of uric acid and reactive oxygen species (ROS), but also triggers oxidative stress. This oxidative stress could intensify the dyslipidemia, exacerbate insulin resistance, and set off a chain of metabolic changes within the body [31, 41]. Concurrently, dyslipidemia could also stimulate an inflammatory response, potentially triggering hyperuricemia, thereby providing a possible explanation for the relationship between lipid profile and uric acid levels [42].

Contrary to popular belief, our research suggests that LDL-C is inversely associated with hyperuricemia [15]. A potential explanation for this counterintuitive relationship could lie in the immunomodulatory functions of LDL-C. Studies have indicated that LDL-C may play a role in immune responses by binding to and neutralizing pathogens and toxins, which in turn could help alleviate the inflammatory responses frequently triggered by hyperuricemia [19, 20, 30]. Moreover, in our comprehensive analysis of non-HDL cholesterol, which encompasses LDL-C and VLDL-C, we observed significant associations with hyperuricemia across increasing quartiles, indicating a potential correlation between elevated non-HDL cholesterol levels and the risk of hyperuricemia [43]. While LDL-C shows a weaker inverse association with hyperuricemia compared to HDL-C, non-HDL cholesterol as a broader category might still provide additional insights into the relationship between dyslipidemia and hyperuricemia, and it should be considered in the clinical assessment of cardiovascular risk.

Most of the previous studies only focused on the relationship between dyslipidemia as a whole or individual indicator and hyperuricemia, but in this study, we found the association between different lipid diseases and hyperuricemia according to the clinical classification of dyslipidemia [24, 44]. According to the results of the study, we found that people with hypertriglyceridemia and mixed hyperlipidemia were more likely to suffer from hyperuricemia. However, people with hypercholesterolemia and low HDL-C are less likely than normal people to develop hyperuricemia. Therefore, doctors should always pay attention to the clinical risk of hyperuricemia in the first group of people.

It is worth noting that the current study has several limitations. First, this study was a cross-sectional study, and we could not determine a causal relationship between hyperuricemia and dyslipidemia. Furthermore, we lack data on factors such as diet and lifestyle that are known to have an impact on the incidence of hyperuricemia. At the same time, we also lack detailed information on uric acid or lipid-lowering drugs that might affect our results. Finally, the relationship between hyperuricemia and dyslipidemia is very complex, and further analysis is needed to more clearly explain the mechanism of influence between the two.

## Conclusion

Hyperuricemia has been shown to have an independent association with dyslipidemia. Furthermore, increased levels of triglycerides or total cholesterol, which are present in conditions like hypertriglyceridemia and mixed hyperlipidemia, are positively correlated with the onset of hyperuricemia.

## Data Availability

The data of participants are collected by the authors and uploaded to the database, which makes it easier for the authors to use the data in the process of analyzing data and writing manuscripts. This kind of database system can conveniently shield the data irrelevant to the experiment and effectively protect the privacy of participants. The data that support the findings of this study are available from the Health Management Center at the First Affiliated Hospital of Wannan Medical College in Wuhu, China but restrictions apply to the availability of these data, which were used under license for the current study, and so are not publicly available. Data are however available from the authors upon reasonable request and with permission of the Health Management Center at the First Affiliated Hospital of Wannan Medical College in Wuhu, China. If someone wants to request the data from this study, they can contact Yufeng Wen (corresponding author).

## Abbreviations

SUA: serum uric acid
BMI: body mass index
FBG: fasting blood glucose
SBP: systolic blood pressure
DBP: diastolic blood pressure
TC: total cholesterol
TG: triglycerides
HDL-C: high-density lipoprotein cholesterol
LDL-C: low-density lipoprotein cholesterol
VLDL: very low-density lipoprotein
non-HDL: non-high-density lipoprotein
ALT: alanine transaminase
AST: aspartate transaminase
BUN: blood urea nitrogen
SCR: serum creatinine
